# The Dead Lion

**Published:** 1831

**Authors:** 


LXII.

THE DEAD LION.

An insignificant correspondent of a contemporary journal for last month, has accused us, while adverting to the eccentricities of the late Mr. Abernethy, of giving a "kick to the dead lion." If we were guilty of this fault, we shall take good care not to commmit a much greater one—that of giving a kick to a living ass—by way of helping him to a little notoriety while braying in return. We are not very prone to censure either the living or the dead; and it would be difficult to assign any plausible cause for our *fear* of Mr. Abernethy in Bedford Row — or our *malice* towards his ashes in the lone church-yard. Those who are acquainted with our writings, know that we freely criticised some of his doctrines and practices when he was well able to reply to our criticisms. Mr. A. never injured or offended us—and if he had done so, we should not have remembered it for 24 hours, much less carried our resentment beyond the grave. But the charge is so puerile—and the object of it is so obvious, that we shall not deign to enter into any serious refutation. Let any of our readers revert to the article, and ask himself if there be any thing severe or unjust in our observations. We surmised that Mr. Abernethy's eccentric coarseness was at first *assumed*, and that afterwards it was continued from *habit*. This is the most charitable interpretation. If such coarseness (many have called it by a worse name) was *not assumed*, the actor must have been a madman or a monster. Utrum horum mavis accipe?

We are charged with the crime of affirming that the routine practice of applying the same remedies to all diseases must have proved injurious to health or even to life in many instances. This we re-assert. More than two years ago we published a striking instance of a life sacrificed by the want of all attention on the part of Mr. A. to the investigation of a disease. A most dangerous disease was going on in the head, and the surgeon would only look at the tongue, strictly prohibiting a word to be spoken by the patient or his wife. The man died at Bayswater of the overlooked disease, and was examined in company with another medical man. This case we published at the time, but Mr. A. never answered it. Yet we are now accused to giving a kick to the dead lion!

---

LXIII.

